# Laparoscopic radical ‘no-touch’ left pancreatosplenectomy for pancreatic ductal adenocarcinoma: technique and results

**DOI:** 10.1007/s00464-015-4685-9

**Published:** 2015-12-16

**Authors:** M. Abu Hilal, J. R. C. Richardson, T. de Rooij, E. Dimovska, H. Al-Saati, M. G. Besselink

**Affiliations:** 1 University Hospital Southampton NHS Foundation Trust, E Level, Tremona Road, Southampton, SO16 6YD UK; 2 Academic Medical Center, Amsterdam, The Netherlands

**Keywords:** Pancreas, Laparoscopy, Surgery, Distal pancreatectomy, Malignancy, Cancer

## Abstract

**Background:**

Laparoscopic left pancreatectomy has been well described for benign pancreatic lesions, but its role in pancreatic adenocarcinoma remains open to debate. We report our results adopting a laparoscopic technique that obeys established oncologic principles of open distal pancreatosplenectomy.

**Methods:**

This is a post hoc analysis of a prospectively kept database of 135 consecutive patients undergoing laparoscopic left pancreatectomy, performed across two sites in the UK and the Netherlands (07/2007–07/2015 Southampton and 10/2013–07/2015 Amsterdam). Primary outcomes were resection margin and lymph node retrieval. Secondary endpoints were other perioperative outcomes, including post-operative pancreatic fistula. Definition of radical resection was distance tumour to resection margin >1 mm. All patients underwent ‘laparoscopic radical left pancreatosplenectomy’ (LRLP) which involves ‘hanging’ the pancreas including Gerota’s fascia, followed by clockwise dissection, including formal lymphadenectomy.

**Results:**

LRLP for pancreatic adenocarcinoma was performed in 25 patients. Seven of the 25 patients (28 %) had extended resections, including the adrenal gland (*n* = 3), duodenojejunal flexure (*n* = 2) or transverse mesocolon (*n* = 3). Mean age was 68 years (54–81). Conversion rate was 0 %, mean operative time 240 min and mean blood loss 340 ml. Median intensive/high care and hospital stay were 1 and 5 days, respectively. Clavien–Dindo score 3+ complication rate was 12 % and ISGPF grade B/C pancreatic fistula rate 28 %; 90-day (or in-hospital) mortality was 0 %. The pancreatic resection margin was clear in all patients, and the posterior margin was involved (<1 mm) in 6 patients, meaning an overall R0 resection rate of 76 %. No resection margin was microscopically involved. Median nodal sample was 15 nodes (3–26). With an average follow-up of 17.2 months, 1-year survival was 88 %.

**Conclusions:**

A standardised laparoscopic approach to pancreatic adenocarcinoma in the left pancreas can be adopted safely. Our study shows that these results can be reproduced across multiple sites using the same technique.

Laparoscopic distal pancreatectomy (LDP) has been shown to be feasible, safe and cost-effective in the management of benign lesions in the body and tail of the pancreas [1–3]. Several meta-analyses have shown excellent results for LDP, at least comparable to those obtained in open surgery [4–9]. However, the validity of the laparoscopic approach when it comes to the management of pancreatic ductal adenocarcinoma is still unclear [10].

Traditional surgical teachings have emphasised meticulous surgical dissection with formal lymphadenectomy and the adoption of ‘no-touch’ techniques in order to obtain a radical oncological resection with minimal risk of tumour dissemination and seeding. These principles have been translated to open distal pancreatectomy for ductal adenocarcinoma by Strasberg et al. [11]. Many would doubt that these high oncological standards can be achieved laparoscopically, and hence, the oncological efficiency of the laparoscopic approach for malignant lesions in the pancreas is still open to debate.

In the absence of a randomised study, data are limited to prospective cohort studies. One series compared results of open and laparoscopic distal pancreatosplenectomy in patients with adenocarcinoma from 9 academic centres over an 8-year period [12]. In that paper, the authors reported on 23 of 212 patients (11 %) who underwent LDP for adenocarcinoma, of which 4 were converted to an open procedure and 4 were completed with a hand-assisted approach. The 74 % R0 resection rate for LDP was not inferior to the 66 % R0 resection rate for open distal pancreatectomy. In this study, the LDP technique used for adenocarcinoma was similar to the technique used for benign disease. Two studies report on the subject of the feasibility of the laparoscopic approach oncologically and showed promising results with 13 case series [13] showing an R1 resection rate of 23 % and a non-inferiority study [14] showing equivalence versus the open approach. Five further selected series [15–19] report on laparoscopic distal pancreatectomy for a mixture of benign and malignant conditions using multiple described techniques including radical en bloc resection in one series preserving the spleen. Between them a total of 56 patients underwent LDP for pancreatic ductal adenocarcinoma. Conversion rates ranged from 0 to 66 %, with surgical margin positivity as low as 0 % and average nodal sample ranging from 6 to 19.8.

We herein report results in performing left pancreatosplenectomy (LRLP) for ductal adenocarcinoma of the pancreas across two university hospitals. We also highlight special tips and tricks that we adopt to ensure a safe and oncologically efficient laparoscopic resection.

## Methods

### Patients

Data on 135 consecutive patients from two university hospitals (105 University Hospital Southampton (UHS: 07/2007–07/2015), 30 Academic Medical Center Amsterdam (AMC: 10/2013–07/2015) undergoing LDP were prospectively collected in a digital database. These included patients undergoing LDP for benign disease and those undergoing LRLP (see ‘Technique’ section) for pancreatic adenocarcinoma. Prospective data collection included: patient demographics, operative details, post-operative details (including complications) and survival.

Procedures were performed by two surgeons over a 7-year period (July 2007 to May 2015) assisted by one or two senior trainees or fellows; the technique was developed at UHS and was also introduced in the AMC after a fellowship at UHS.

All patents were imaged pre-operatively using computed topography (CT) of the abdomen and pelvis. Where malignancy was suspected, formal multidisciplinary team (MDT) evaluation was undertaken (including CT, endoscopic ultrasound and magnetic resonance imaging (MRI) if needed). Biopsy was not performed for solid lesions to avoid seeding of malignant cells. All patients with localised left-sided disease were treated laparoscopically, even if this required adrenalectomy or small bowel resection. Patients were not considered for laparoscopic surgery if the MDT consensus was that the patient required an extended multivisceral resection, requiring colonic, renal or gastric resections or mesenteric vein resection which could not be performed laparoscopically underwent open surgery.

When indicated patients were assessed intra-operatively with laparoscopic ultrasound (see ‘Technique’ section).

Only patients undergoing LRLP for adenocarcinoma of the pancreas, confirmed on post-operative histopathology, were included in the study and therefore used in the analysis.

### Definitions

Complications were classified using the Clavien–Dindo score [20]; minor complications were considered grade I–II, and major complications were considered to be anything scored III or greater. Only clinically relevant (grade B/C) ISGPF grade post-operative pancreatic fistula were registered [21].

Margin status was determined at histopathological examination of the resected specimens in all cases. Specifically by assessing the tumours proximity to all surfaces of the gland, including anterior, posterior and staple line where an R0 resection represented tumour >1 mm from the resection margin or pancreatic surface, R1 resection represented tumour ≤1 mm from the resection margin or pancreatic surface (not as in previous studies: microscopically negatively involved resection margin) and R2 resection represented a macroscopically positive margin [22]. All specimens were further examined to determine both lymph node yield and positivity to provide an accurate staging of the resected tumour using the TNM American Joint Committee on Cancer (AJCC) 7th edition [23].

### LRLP operative technique

**Fig. 1:**
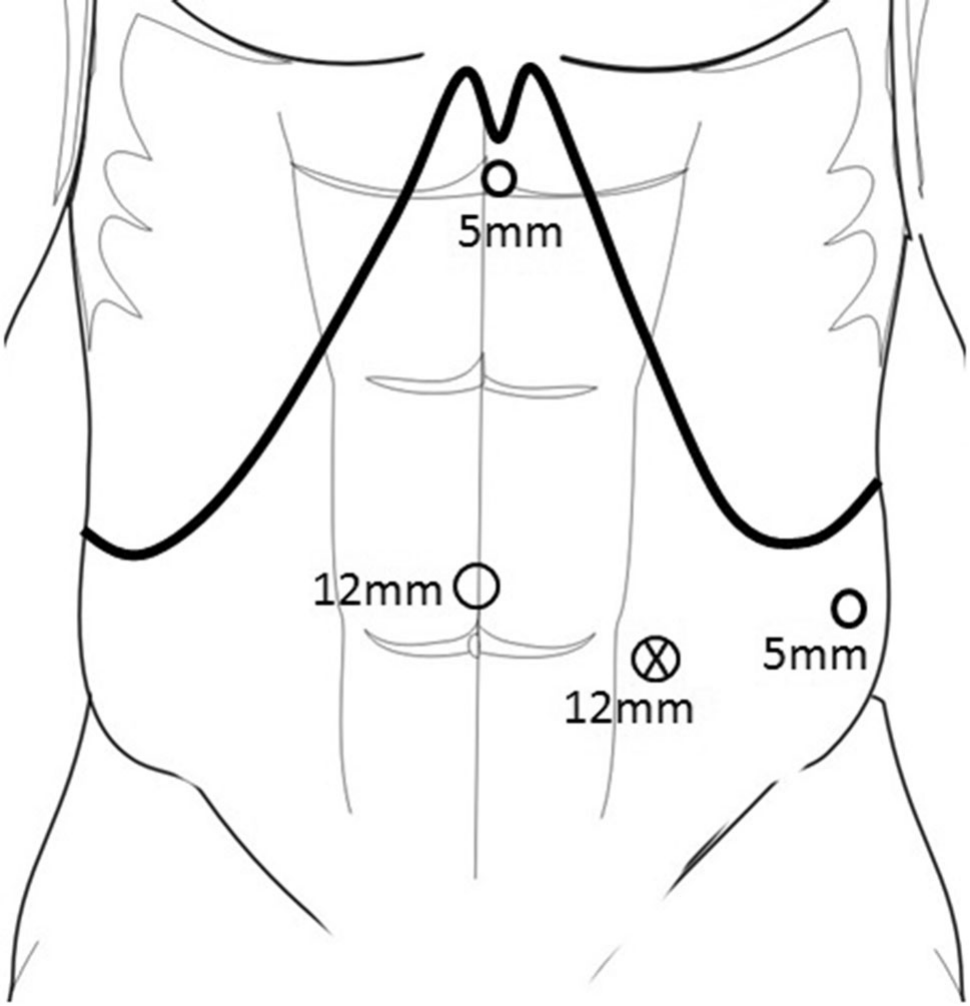
Port placement

Patients are positioned supine with a wedge under their left side to achieve a 30° tilt. In our experience, a tilt of more than 30° can make the access to the neck of the pancreas and the coeliac trunk more difficult. After open sub/supra-umbilical cut-down, a 12-mm trocar is inserted and pneumoperitoneum established. Further 3 trocars are inserted under vision; one 5 mm high in the epigastrium, a 5 mm at the left anterior axillary line, 3–5 cm under the costal margin, and a 12-mm port between the umbilical and left flank port (Fig. 1). Trocar position should be adapted to both the size of the patient and the location of the tumour (body or tail).

Dissection is performed using a combination of diathermy hook, ultrasonic dissector (Harmonic ACE, Ethicon EndoSurgery, Cincinnati, OH, USA, or Lotus Laparoscopic Dissecting Shears, SRA Developments Ltd, Devon, UK), 5 mm or 10 mm clip applicators (Ligamax/Ligaclip, Ethicon EndoSurgery) and, for vessels larger than 7 mm, an endoscopic ligation system (Hem-o-lok, Teleflex, NC, USA).

A Nylon tape 3 × 70 mm (Ethicon Nylon Tape Ethicon EndoSurgery), one red and one blue sling 2 × 70 mm (Silicone Sling, DTR Medical, Swansea, UK) are each divided in four and used for the hanging and slinging manoeuvres during the procedure (red slings for arteries, blue slings for veins and the nylon tape for the pancreas). We prefer short lengths as this is enough for the purpose, preventing multiple slings and tapes disturbing the operative field, and allows easier passage through a 5-mm port.

#### Step 1: Access and exposure

Following diagnostic laparoscopy, the gastrocolic ligament and short gastrics are divided and a monofilament non-absorbable suture is passed twice through the posterior gastric wall. This suture is externalised via the fascial opening used for the epigastric port, thus retracting the stomach and exposing the pancreas and the lesser sac. The spleno-colic ligament is divided, and the splenic flexure mobilised to permit a complete exposure of the pancreatic tail. Laparoscopic ultrasound is performed if needed to determine tumour location or extension. If the splenic artery is visible, it is dissected, slung and occluded using a laparoscopic bulldog applicator until a definitive division of the splenic artery is achieved. This manoeuvre reduces pancreatic and splenic vascularisation and therefore blood loss during the dissection.

#### Step 2: Gerota’s dissection and pancreatic hanging

**Fig. 2:**
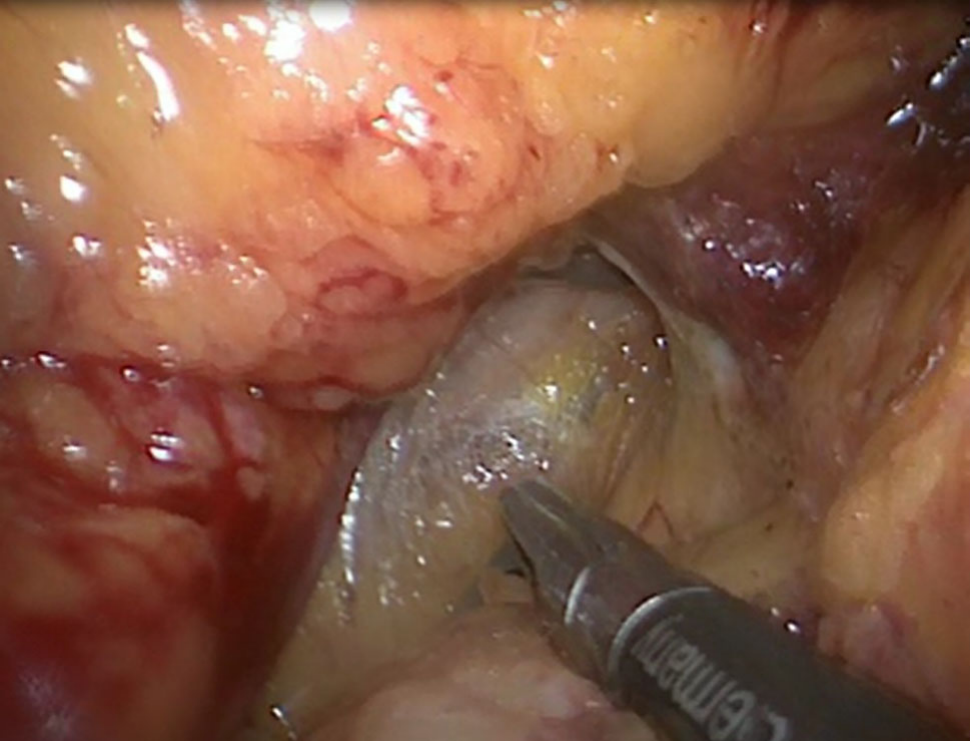
Mobilising Gerota’s fascia caudo-cranial direction

**Fig. 3:**
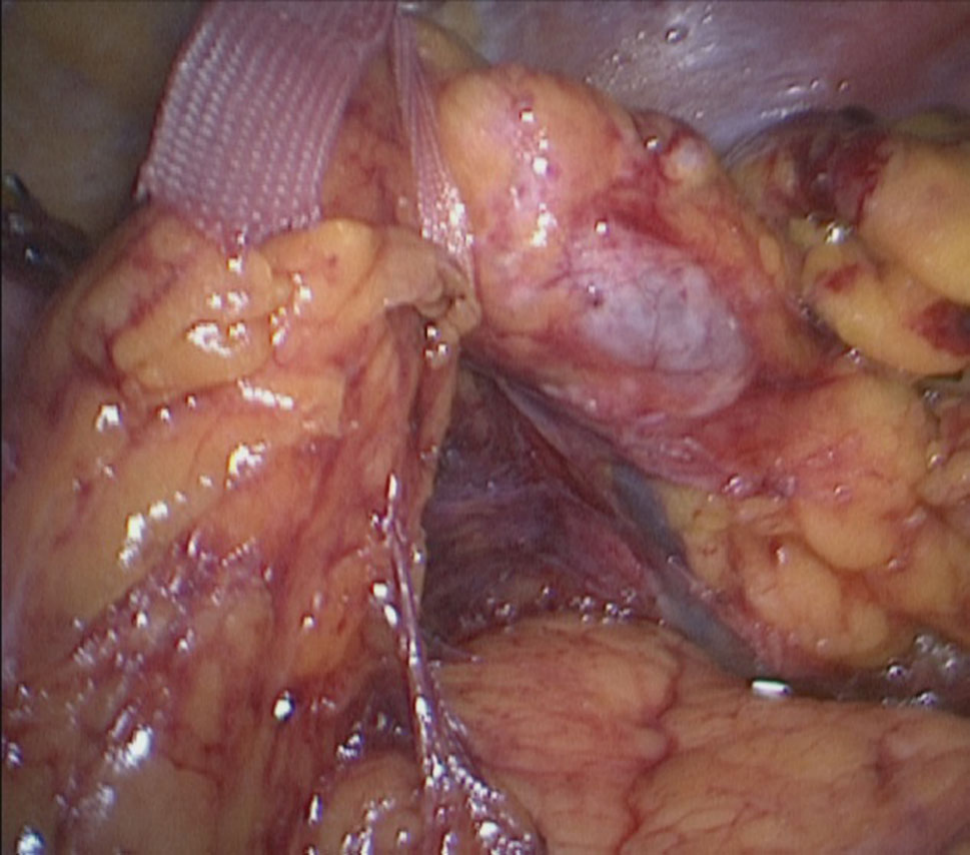
Slinging of pancreas with nylon tape

The inferior border of the pancreas is dissected in an area distant from the neoplasm (normally distal unless the lesion is in the tail). The mobilisation continues down until Gerota’s fascia (or: renal fascia) is identified, which is then incised and lifted (Fig. 2). The posterior plane is developed from here between this and the adrenal gland towards the superior pancreatic margin. The superior margin is then dissected and Gerota’s fascia is incised at the same level, thus joining the dissection from below. A soft grasper is passed through this developed plane inferiorly until the tip is seen from the superior margin and a nylon tape is then pulled through and secured with an endoscopic clip (Hem-o-lok, Teleflex, NC, USA), thus allowing the pancreas to be ‘hung’ (Fig. 3). The dissection of the inferior margin and the development of the posterior plane are continued clockwise towards the splenic/superior mesenteric vein junction. A second tape is passed under the pancreas to the right of the neoplasms to help in lifting the pancreatic body, offering adequate tissue tension and creating a clear dissection plane from the retroperitoneum.

#### Step 3: Vessels

**Fig. 4:**
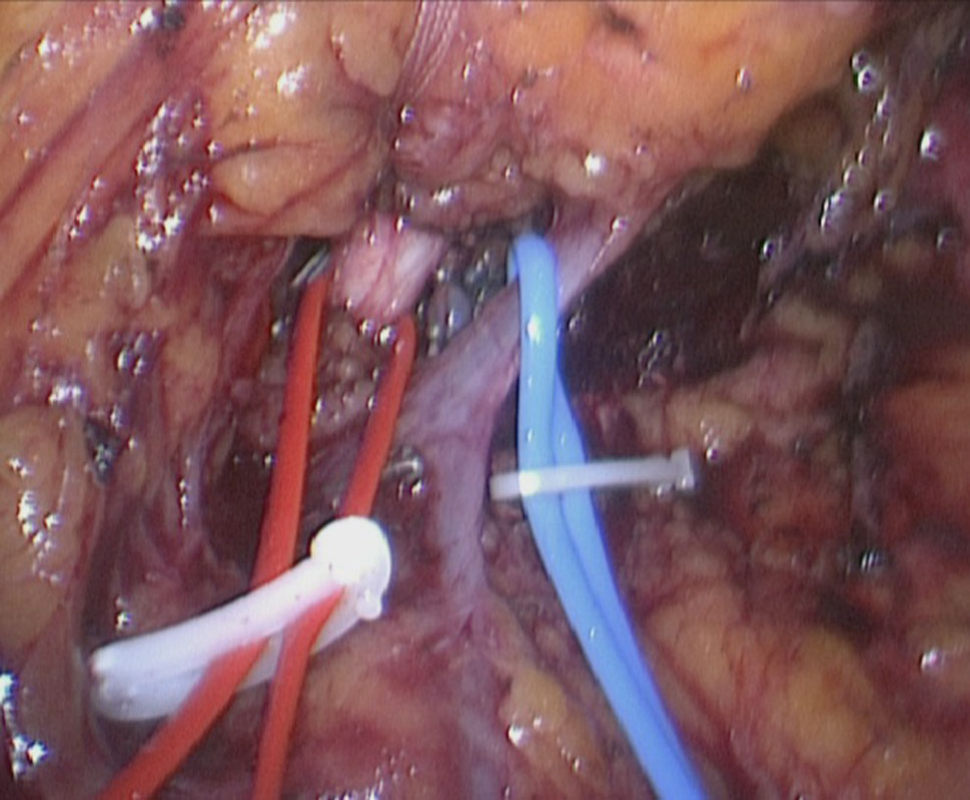
Slinging of splenic artery and vein

The tunnel under the pancreatic neck plane is then developed using a combination of blunt dissection and, if needed, hydrodissection with a regular laparoscopic suction device. In some cases, the hepatic artery is dissected and slung to permit the passage of a third nylon tape around the neck of the pancreas. Lifting the pancreas with the two medial tapes offers an excellent view of the pancreas and the posterior vascular structures. The splenic vein is dissected, slung (Fig. 4) and secured with two endoscopic clips (Hem-o-lok, Teleflex) at its junction with the superior mesenteric vein, then divided. Depending on the relation to the tumour, the inferior mesenteric vein is transected or left intact.

#### Step 4: Pancreatic transection

The second part of the dissection is in a medial–lateral direction. The pancreas is transected at the neck keeping a clear margin from the lesion, using a linear stapler device (Echelon 60, Ethicon EndoSurgery) with a vascular cartridge employing a slow compression technique allowing approximately 120 s for complete closure of the stapler with an aim to prevent rupture of the pancreatic capsule.

#### Step 5: Lymphadenectomy

**Fig. 5:**
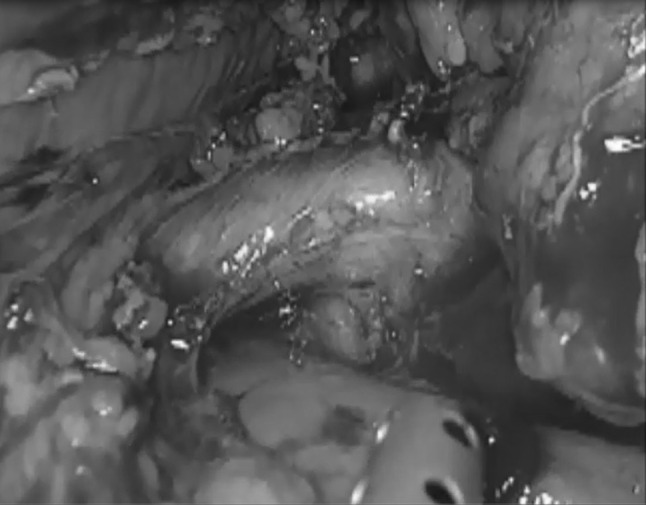
Lymphadenectomy common hepatic artery

The pancreas is then dissected from the hepatic artery and the coeliac trunk performing a full nodal clearance including station 8, hepatic nodes (Fig. 5). The origin of the three coeliac vessels is seen, and nodal clearance is performed down to the coeliac trunk and inferiorly on the left border of the aorta to the left of the superior mesenteric artery; the splenic artery is slung (if not done before) and secured with three endoscopic clips (Hem-o-lok, Teleflex) and divided at its origin. If needed the left gastric artery is slung and lifted to help in completing the nodal clearance around it. The clockwise dissection is continued towards the spleen, taking any remnant attached tissue and nodes including the Gerota’s fascia.

#### Step 6: Extraction, closure

The spleen is then released from its remaining retroperitoneal attachments. The specimen is removed from a Pfannenstiel incision (approximately 6 cm) in an impermeable extraction bag (Endocatch, Ethicon EndoSurgery) that was passed through a 15-mm port. Care is taken not to crush the specimen in order to compromise pathological assessment. The peritoneum is closed with one vicryl and the fascia with a one PDS loop, and this is then visualised laparoscopically to ensure optimal closure. The resection site is inspected and haemostasis ensured. The stump is only secured with interrupted PDS 3/0 sutures if there are areas of parenchymal fractures or ongoing bleeding. A combination of absorbable haemostatic material (SURGICEL SNoW, Ethicon Endosurgery) and haemostatic glue (EVICEL Fibrin Sealant (Human), Ethicon Endosurgery) can be applied. A 20F Wallace drain is placed adjacent to the resection line of the pancreas with a loop in the splenic bed with 2–3 additional side holes at this site and secured. The fascial defects on all ports larger than 5 mm are closed using an absorbable multifilament suture and skin closed using absorbable monofilament.

## Results

### Patient demographics and operative details

Of the 135 patients undergoing LDP during the study period, 25 patients (18.5 %) underwent LRLP for adenocarcinoma of the pancreas (20 at UHS and 5 at AMC). Of these, 24 patients were suspected as adenocarcinoma pre-operatively and underwent planned LRLP following laparoscopic assessment and in 1 patient a radical resection was performed based on intra-operative findings (this patient had background chronic pancreatitis and an uncertain diagnosis pre-operatively). Seven of the 25 patients had extended resections, and these involved subtotal left adrenal gland resection (*n* = 3), duodenojejunal flexure wedge resection (using an endoscopic stapling device) (*n* = 2) and partial resection of the transverse mesocolon (not involving the colon itself) (*n* = 3).

Mean age was 68 years, and 48 % of patients were male. All cases were completed via a pure laparoscopic technique (i.e. no use of hand ports) with a 0 % conversion rate. Mean operative time was 240 min (range 120–390 min). Average blood loss was 340 ml (range 50–1000 ml), and none of the patients required intra-operative or post-operative blood transfusion. These findings are summarised in Table 1. During the study period, 5 patients underwent open resections 3 at UHS (all due to scheduling difficulties and surgeon non-availability at the time) and 2 at AMC (1 due to tumour involving the celiac axis requiring reconstruction and 1 had an exploratory open procedure to establish respectability in which they proceeded).

**Table 1:** Patient demographics and intra-operative results

Demographic	*n* = 25
Age (years)	68 (54–81)
Male (%)	48 %
Conversion rate (%)	0 %
Operating time (min)	240 (120–390)
Blood loss (mL)	340 (50–1000)
Intra- or post-operative blood transfusion (units)	0

### Post-operative details

Median hospital stay was 5 days (range 2–57 days). Patients stayed a median of 1 day in intensive care or high dependency unit (range 0–27 days). Median ward stay was 3 days (range 1–30 days). An enhanced recovery programme was introduced during the series which brought hospital stay down to as low as 2 days [24]. Five patients were readmitted within 30 days (1 with nausea and vomiting, 2 with peripancreatic collections and 2 to manage post-operative pancreatic fistulae). Two patients (8 %) required radiological drainage for infected peripancreatic collections, and 11 patients developed ISGPF post-operative pancreatic fistulae (44 %; of any grade) with seven of these patients (28 %) developing a ISGPF grade B fistula meaning that they were discharged with the surgical drain in situ. This was managed by serially withdrawing the surgical drain at outpatient visits. The 90-day mortality was 0 %. The average follow-up was 17.2 months in that time there were 6 mortalities at 2 at 6 months, 1 at 11 months, 1 at 12 months, 1 at 15 months and 1 at 43 months post-operatively, leading to an overall survival of 76 % and a 1-year survival of 88 %. These findings are summarised in Table 2.

**Table 2:** Post-operative results

Days on ITU/HDU (days)	1 (0–27)
Ward stay (days)	3 (1–30)
Total hospital stay (days)	5 (2–57)
Clavien–Dindo ≥3 complication rate (%)	12
Radiological post-operative intervention (%)	8
ISGPF grade B/C fistula rate (%)	28
Readmission rate (%)	20
90-day mortality (%)	0
Follow-up (months)	17.2 (±16.5)

*ICU* intensive care unit, *HDU* high dependency unit

### Histopathology details

All patients were shown to have ductal adenocarcinoma of the pancreas upon histopathological assessment of resected specimens, and these findings are summarised in Table 3. Of these, 2 patients (8 %) were found to have T4 disease (both N1), 21 (84 %) found to have T3 disease, 1 (4 %) found to have T2 and 1 (4 %) found to have T1 disease. Eighteen patients (72 %) had N1 disease, and no patients had N2 disease.

**Table 3:** Histopathology

T1	1 (4 %)
T2	1 (4 %)
T3	21 (84 %)
T4	2 (8 %)
N0	7 (28 %)
N1	18 (72 %)
N2	0 (0 %)
Tumour size (mm)	36 (15–82)
Total nodes (range)	15 (3–26)
Positive nodes	2 (0–5)
Specimen length (mm)	100 (70–160)
Retroperitoneal margin positive	24 %
Multivisceral resection	6 (24 %)

Average tumour size was 36 mm (range 15–82 mm) with median nodal sample of 15 (range 3–26 nodes) of which a median of 2 positive nodes were found (range 0–5 nodes). The staple margin was free of tumour in all patients. Six patients (24 %) had positive posterior ‘margins’ as tumour was found less than 1 mm from the surface, of these there was no microscopic involvement of tumour in the staple margin or posterior margin, and these patient characteristics are summarised in Table 4. Ten patients demonstrated perineural invasion, and ten patients had signs of vascular invasion with one of these demonstrating tumour thrombus in the splenic vein. Upon microscopic examination of the specimens of extended resections [adrenal gland (*n* = 3), duodenojejunal flexure (*n* = 2), mesocolon (*n* = 3)], margins were found to be adequately clear.

**Table 4:** Retroperitoneal margin positive tumour characteristics

Survival (months)	Size (mm)	pTN	Nodal involvement	Retroperitoneal margin (mm)	Perineural invasion	Vascular invasion	Infiltration of adjacent organs
6 (Dead)	15	T3N0	0 of 3	0.5	Yes	No	No
12 (Dead)	50	T3N0	3 of 21	<1	Yes	No	No
28 (Dead)	30	T4N1	2 of 14	<1	No	Yes	Yes
6 (Dead)	45	T3N1	3 of 26	<1	No	Yes	Yes
5 (Alive)	25	T3N1	1 of 10	0.7	No	No	No
11 (Dead)	29	T3N1	3 of 20	<1	Yes	Yes	No

## Discussion

This study described the results of laparoscopic left pancreatosplenectomy exclusively for pancreatic ductal adenocarcinoma in two university hospitals in the UK and the Netherlands. Based on the results in 25 patients, we concluded that, in selected patients, the standardised LRLP approach to pancreatic adenocarcinoma in the left pancreas can be adopted and reproduced with results equivalent to those obtained in historical open series.

Previous studies have grouped results of LDP for malignant and benign conditions [4–9], hampering specific analyses of the laparoscopic management of pancreatic ductal adenocarcinoma. Of the studies that report specifically on LDP for pancreatic cancer, Lee et al. [18] did so in a highly selected group of patients (tumours confined to the pancreas), and whilst reporting excellent results their outcomes are not applicable to the treatment of all patients with left-sided cancers. Kawaguchi et al. [19] also showed promising results though their technique departs widely from established oncosurgical practice since they preserved the spleen in 17 of 24 patients [11].

A recent report from the National Cancer Database in the USA [25] included patients from 1500 hospitals between 2010 and 2011. This retrospective study reported on 144 patients undergoing laparoscopic distal pancreatectomy for ductal adenocarcinoma, and of these, there was 12 % margin positivity with a median nodal sample of 13. They compared these outcomes with patients undergoing open distal pancreatectomy during the same time period and found equivocal oncological outcome measures.

Oncological surgery requires a radical resection, adequate lymphadenectomy and meticulous ‘no-touch’ dissection as it may prevent seeding and tumour cell dissemination [26, 27]. The oncological approach to tumours of the body and tail of the pancreas has been well described in open surgery [11, 28–31]. This involves the division of the pancreas at the neck, removing the pancreatic body and tail, including Gerota’s fascia (as stressed by Strasberg [11]) using a medial-to-lateral approach. The resection may include the adrenal gland in case of tumour extension. Strasberg described the oncological lymphadenectomy based on the reviewed concepts of lymphatic drainage of the pancreas described by O’Morchoe [32]. The oncological approach to these tumours is reported by Fernández-Cruz et al. [13] who describe a laparoscopic variant of Strasberg’s radical antegrade modular pancreatosplenectomy on 13 patients with pancreatic adenocarcinoma, and they showed an R1 resection rate of 23 % in this group with an average nodal sample of 14.5. A study from Pittsburg, USA [14], involving 28 patients undergoing LDP and 34 having open surgery both for adenocarcinoma over an 8-year period showed non-inferiority of the laparoscopic approach in their analysis specific to oncological outcome measures.

In LDP for benign disease, the pancreas is usually divided just proximally to the lesion. Routine division at the pancreatic neck is not mandatory and not the best option to preserve parenchyma. This is different in oncological resection for cancer, where the whole left pancreas should be removed in order to obtain a radical resection clearing all the lymphatic stations and Gerota’s fascia. This is why we elect to call our resection left pancreatectomy differentiating it from the distal pancreatectomy.

Our described technique for LRLP takes into consideration all the above. Hanging the pancreas at different levels allows for ‘no-touch’ dissection, keeping the planes under tension, whilst offering excellent retropancreatic views. Slinging the vessels permits a better understanding of the anatomy before dividing any vital structures but also facilitates nodal clearance around and between the vessels.

Only a few series have clearly reported on margins and nodal clearance after open distal pancreatectomy for pancreatic ductal adenocarcinoma. The previously mentioned multicentre study [12] comparing laparoscopic and open distal pancreatectomies for adenocarcinoma in 39 patients reported no difference in R1 resection rate between laparoscopic (26 %) and open (34 %). R1 was defined as a microscopically involved margin and not as a <1-mm free margin. A single-centre study [15] including 18 patients undergoing LDP for ductal adenocarcinoma reported a 3 % R1 resection rate. In this study, R1 was defined as a microscopically involved resection margin. Our results compare favourably to these studies; if we were to use the same (old) definition, the current series has a 4 % R1 resection rate. However, we believe that R1 resection should be defined as <1 mm from the transection or the posterior margin as recommended by Verbeke et al. [21]. Even when we apply this definition, our R1 rate is comparable to previously reported outcomes in open surgery. The factors contributing to causing a positive margin are beyond the scope of this study, but in analysing our patients it can be observed that all are T3 and T4 disease and lie on the posterior surface of the pancreas.

A recent analysis by Baker et al. [33] concluded that LPD failed to provide a lymphadenectomy comparable to open distal pancreatectomy as only five lymph nodes were retrieved with the laparoscopic approach versus nine with the open approach. The previously mentioned multicentre analysis by Kooby et al. [12] reported on a mean nodal sample of 13.8 nodes. Our histopathological results (median nodal sample 15, range 3–26 nodes, mean tumour size 3.6 cm) are comparable with series on open distal pancreatectomy with median nodal sample 15 and tumour range between 2 and 3.6 cm [12, 15–17]. Although the advantage of a formal lymphadenectomy in distal pancreatectomy has not been proven, this cannot be an excuse for suboptimal lymphadenectomy during a laparoscopic approach to pancreatic ductal adenocarcinoma.

Our hospital stay of 5 days, mean blood loss of 340 ml and operative time of 240 min also compare favourably to large open series, such as reported from Johns Hopkins [30] with average hospital stay of 9 days, mean blood loss of 912 ml and operative time of 300 min in 235 open distal pancreatectomies including 43 for pancreatic ductal adenocarcinoma.

Our study reports a 0 % conversion rate with a pure laparoscopic technique, despite the extensive surgery required in some cases. This was due to tumour size and invasion of adjacent organs such as the adrenal gland, the mesocolon and duodenal jejunal flexure which required resection in 2 cases. In the five previously mentioned studies on LDP in adenocarcinoma, the mean conversion rate across all studies was 22 % (range 0–66 %). A large series from the Memorial Sloan Kettering Cancer Center reported on 343 distal pancreatectomies over a 7-year period. Only 18 patients underwent a LDP for a malignancy. The conversion rate in all 107 laparoscopic procedures was 30 % [17]. No data on radicality in the laparoscopic procedures for malignancy were provided.

The current study is primarily limited by its small numbers. However, 25 cases completed fully laparoscopically compares favourably with 15 procedures  completed laparoscopically in a multicentre study including nine centres [12]. Retrospective studies carry a risk of selection bias. Notably, our study has included all comers regardless of size and location of the tumour. In addition, we have included tumours which needed multivisceral resections to achieve a radical clearance, minimising potential selection bias. In addition, all cases were performed by one of the two laparoscopic pancreatic surgeons using the same standardised approach.

Last but not least, in addition to safety, feasibility and oncological efficiency, a surgical technique must be reproducible, easy to teach and easy to ensure validity and expansion. Our report represents an excellent example of how after a year of fellowship and mentoring (at UHS), one of our surgeons was able to safely establish LRLP in his centre, adopting the same technique and achieving similar results. We believe our described technique can be useful for surgeons who are starting the laparoscopic approach in their centres as most of the technical items explained can be useful in LDP for benign disease as well.

Our study suggests that where sufficient expertise with laparoscopic resections for benign pancreatic conditions is available [2], LRLP can be used as a treatment for pancreatic lesions regardless of aetiology. Where malignancy is suspected, a ‘no-touch’ technique following principles used for radical open surgery should be adopted, and this can be done so using our approach described across multiple sites. This technique can be used in robotic resections in future practice, as it is based on established oncological principles which remain relevant regardless of surgical approach. Further work should focus on long-term oncological outcome of this procedure and larger multicentre studies focusing solely on LRLP for pancreatic ducal adenocarcinoma. This indication could represent an ideal proposal for a randomised controlled multicentre trial.

## Abbreviations

LDP: Laparoscopic distal pancreatectomy
LRLP: Laparoscopic radical ‘no-touch’ left pancreatosplenectomy
UHS: University Hospital Southampton, Southampton
AMC: Academic Medical Center, Amsterdam
ISGPF: International Study Group Definition on Pancreatic Fistula
CT: Computed topography
MDT: Multidisciplinary team
MRI: Magnetic resonance imaging
